# Motoric Cognitive Risk Syndrome: Predictor of Dementia and Age-Related Negative Outcomes

**DOI:** 10.3389/fmed.2017.00166

**Published:** 2017-10-25

**Authors:** Jagadish K. Chhetri, Piu Chan, Bruno Vellas, Matteo Cesari

**Affiliations:** ^1^Gérontopôle, Department of Geriatrics, Centre Hospitalier Universitaire de Toulouse, Toulouse, France; ^2^Department of Neurobiology, Geriatrics, and Neurology, Beijing Institute of Geriatrics, Xuanwu Hospital of Capital Medical University, Beijing, China; ^3^INSERM UMR 1027, University of Toulouse III Paul Sabatier, Toulouse, France

**Keywords:** dementia, motoric cognitive risk, gait, cognition, subjective memory complaint, geriatric disorders

## Abstract

Cognitive disorders represent a leading cause of disability in the aging population, of which dementia has the highest global burden. Early signs of dementia such as slow gait and memory complaints are known to present well before the overt manifestation of the disease. Motoric cognitive risk (MCR) syndrome characterized by the simultaneous presence of gait disturbances and memory complaints in older subjects has been proposed to study the close interactions between the physical and cognitive domains as well as a possible approach to identify individuals at increased risk of dementia. In addition, studies have shown MCR as a predictor of other negative outcomes in older adults, including disability, falls and death. However, the concept of MCR is still in its early stage and approach to the syndrome is still not well established. This review aims to put together the various aspects of MCR syndrome including its pathophysiology, diagnosis, epidemiology, and relationship with other geriatric conditions.

## Introduction

Older adults are known to have decreased functional capacity (e.g., sensory, cognitive, physical), which makes them vulnerable to adverse events such as disability, dependency, falls, or even death (1–5). Poor mobility of lower limbs with aging is one of the most commonly presented form of physical limitation in older individuals (6). Several studies have shown gait speed to predict major health-related events in older adults (6–8). Similarly, decline in memory is another common form of cognitive limitation associated with increase in age, which might potentially progress to dementia (9, 10). Furthermore, evidences from past studies have shown coexistence of cognitive decline and gait abnormality (that might be of musculoskeletal or neuro-sensory-motor etiology) to be a common condition in older adults (3, 11, 12). Besides, these functional limitations are known to be the major causes of disability and dependency in older adults (7, 11, 13, 14).

Growing body of evidence suggests that simultaneous presence of cognitive complaints with reduced gait speed may indicate early signs of dementia (presenting decades before actual presentation of cognitive impairment) (15–20). Unfortunately, very little is known about how the actual interaction between the cognitive and physical domain (such as which domain triggers the other, or time-point of initiation) occurs with the phenomenon of aging. Intuitively, an entity that captures both physical and cognitive functional status of an aging individual could reflect a more implicit functional status of the individual. Moreover, such entity would aid researchers to better understand the interaction between cognition and physical domains in aging individuals who are at high-risk of dementia and other geriatric disorders.

In this review, we discuss on a novel concept described as motoric cognitive risk (MCR) syndrome that captures the state of concomitant presence of gait disturbances and cognitive decline in older adults (20). Studies have shown MCR to be an effective tool in predicting various geriatric conditions such as dementia (19), falls (21), disability (22), and mortality (1).

## Gait and Cognition in Older Adults

*Gait*: Walking is a very common activity of daily living, which at a glance appears to be an entirely unsophisticated automated motor task. However, maintaining of normal gait is a much complex process requiring intact multisystem (nervous, sensory, musculoskeletal, cardiorespiratory) function and coordination (3, 16, 17). With increase in age, the parameters of gait (velocity, stride length, swing time) are affected as a result of disturbances in either of the musculoskeletal functions, locomotor function, balance, postural reflexes, sensory function and sensorimotor integration, and cardiorespiratory functions (23), resulting abnormal gait.

At present, gait speed or gait velocity has increasingly been implemented in clinical settings to evaluate functional status in older subjects and even to predict adverse events (1, 18, 21, 22, 24). In addition, slow gait speed is thought to be a sensitive marker of cognitive decline with aging (18, 25–28). However, the methods and cut-off values for assessment of gait in older subjects is known to vary widely. Moreover, every cut-point might be arbitrary because the relationship between gait speed and risk of negative outcomes follows a linear trend. Nevertheless, gait speed less than 0.8 m/s over a 4-m track is one of the most commonly used cut-points to assess gait speed in older subjects (29). The cut-point has been suggested to predict adverse events in older adults by the International Academy on Nutrition and Aging task force (29) and recommended for further clinical investigation by the European consensus on sarcopenia (30). On the other hand, the concept of “dis-mobility” describes a much slower gait speed of less than 0.6 m/s to be a relevant cut-point suitable for improving clinical care, research, and regulatory approval of treatments to improve mobility in older adults (31). Gait abnormalities have been identified from early neurological studies and subclassified as unsteady, ataxic, frontal, parkinsonian, neuropathic, hemiparetic, or spastic (23, 32) depending upon the nature of the disturbances that should be properly identified by physicians while assessing older adults.

*Cognition*: Cognition relates to the functioning processes of the brain, which tends to change with age (33). Cognitive functions such as attention, intelligence, memory, processing speed, and executive function are known to decline with increase in age with varying degree between individuals (34), which could affect the overall functioning of an individual including gait. This alteration in cognition with aging has been associated primarily with decline in brain gray and white matter volume (35), brain hippocampus volume (36), and deposition of protein beta-amyloid in brain [a primary marker of Alzheimer’s disease (AD)] (37). Factors such as cardiovascular diseases (and associated risk factors), genetics, low level of education and depression have been identified as major contributing factors for cognitive decline (38), which could simultaneously influence the overall physical functioning. Assessment scales such as clinical dementia rating (CDR) (39), Mini-Mental State Examination (40), and other forms of dementia screening questionnaire have been widely used to assess overall cognitive status of older adults.

### Link between Gait and Cognition

As discussed before, gait is a sequel of multifactorial and multisystem coordination, but primarily the result of neuromuscular interaction capacity of an individual. Anatomically speaking, brain frontal subcortical circuits predominantly mediate gait (41). Executive function (3, 42) and attention (42–44) have been suggested to be the primary cognitive processes associated. However, the frontal lobe itself is vulnerable to age-related changes (42), which could alter gait speed and cognition in older adults. In addition, declination or improvement in executive function and attention over time was found to effect gait progression (speed) in older adults (17, 27, 42). Increased brain subcortical white matter hyper-intensities (leukoaraiosis) (45) and decrease in cerebellar gray matter volume (46) and hippocampal volumes (47) were found to be associated with reduced gait speed. This overlap between the brain areas controlling gait and cognition explains the relationship between slow gait and dementia pathologies. Furthermore, a dual-task methodology termed as “walking while talking” was developed for making the gait speed assessment more challenging and included the evaluation of cognition (48–50). The participant’s change in motor performance during dual-task was observed suggesting requirement of additional cognitive resources to maintain multisystem coordination, which might be difficult to achieve for older individuals with cognitive limitation, leading to detrimental effects such as falls. Besides, a recent meta-analysis has shown evidences of brain structure to be associated with muscle structure and function (51), showing the consequential association between these domains, which could alter gait function.

#### Factors Associated

Physical limitations and cognitive decline have been suggested to present bidirectional relationship (3, 17, 52). These conditions most likely share the common risk factors and pathways such as chronic inflammation, hormonal pathways, lifestyle factors, and even genetic pathway (11, 53–57).

Low-grade chronic inflammation or “inflamm-aging” might be the primary biological pathway shared by gait and cognition in older individuals (58–62). Atherosclerosis, a chronic inflammatory condition in older adults is known to promote cardiovascular dysfunction that could increase functional loss (both cognitive and physical) in aged individuals (61, 63, 64). Furthermore, chronic inflammation is found to directly impact the central nervous system (e.g., neurofibrillary tangles, amyloid plaques) of older adults (65–67) and promote cardiovascular risk factors (59, 61, 68, 69). Increased serum C-reactive protein, interleukin-6, and plasma tumor necrosis factor-α are the inflammatory markers associated with decrease in total brain volume (58, 65, 70, 71) that could affect cognition and gait simultaneously.

Cardiovascular risk factors (e.g., hypertension, diabetes, hyperlipidemia) are known to enhance incidences of cerebral ischemia affecting the periventricular white matter (64, 72, 73). As explained before, brain white matter plays an important role in the control of gait and cognitive processing and responsible for executive function (45, 64, 73). Similarly, other conditions such as neurodegeneration (e.g., in Parkinson’s disease) in older adults is well known to impact both cognition and motoric functions (74).

Needless to elaborate, nutritional factor is a key component to influence physical function in humans. Besides, abundant studies have shown that the deficiency of nutritional factors may affect both cognition and physical functions in older adults (53, 54, 75, 76). Similarly, physical exercise is another factor that is well known to influence both cognition and physical limitations in older adults (77–80). Functional decline in older adults is also known to be influenced by hormonal alteration (such as downregulation of insulin-like growth factor) with aging (81) and genetic factors such as apolipoprotein-E4 (APOE-4 genotype) (55, 56).

## MCR Syndrome

**Figure 1:**
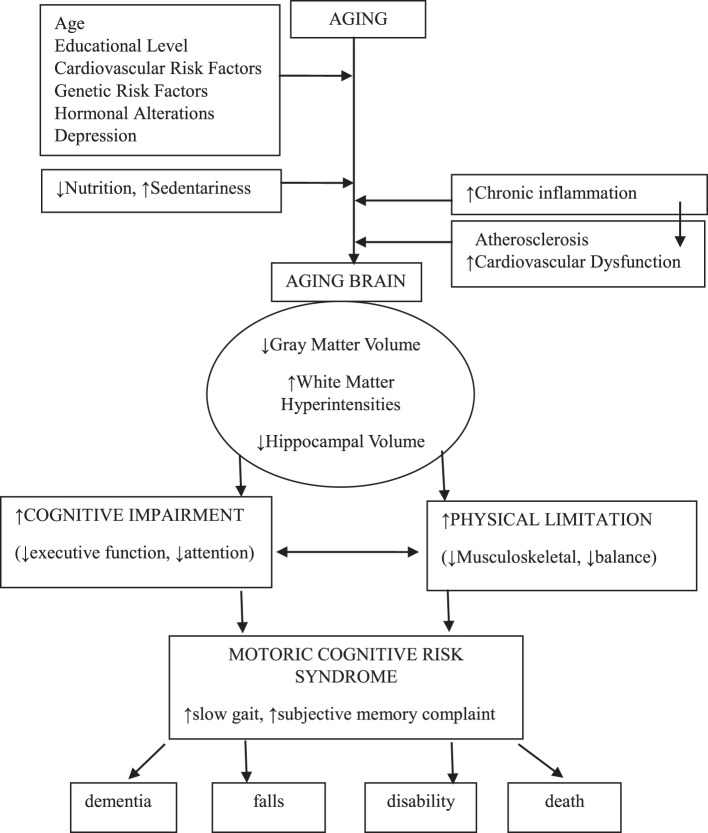
Diagrammatic presentation of motoric cognitive risk syndrome.

Motoric cognitive risk syndrome is defined as a condition characterized by slowness of gait in the presence of subjective cognitive complaint in older adults without any form of dementia or mobility disability (1, 18–22). The theory that slowness of gait coexisting with cognitive decline might be an early sign of dementia, which has been used in this novel entity, potentially resembling a pre-dementia syndrome (18, 19, 22) (Figure 1).

The following four criteria have been proposed to be met for the diagnosis of MCR (although the use of scales was not uniform in prior studies—Table 1) (1, 18–22): (1) presence of subjective cognitive complaints, assessed using standardized questionnaire (e.g., CDR, GDS, or AD screening questionnaire), (2) presence of slow gait: defined as velocity one SD or more below age- and sex-appropriate mean values, (3) preserved mobility, and (4) absence of dementia.

**Table 1:** Frequent methods implemented to diagnose motoric cognitive risk syndrome.

Study	Setting	*N*	Age	Assessment of diagnostic criteria			
				Cognitive complaints	Slow gait	Preserved mobility	Absence of dementia
Verghese et al. (19)	CS	997	≥70 years	CERAD questionnaire	Instrumented walkway (GAITRite)	Preserved ADL assessed by a scale developed for assessing function in community-residing older adults (82) + clinician’s interviews	DSM-IV

Verghese et al. (83)	MC	26,802	≥60 years	Self-report cognitive questionnaire, GDS*, GDS and AD8, IADL	Instrumented walkway (GAITRite)/timed walk (over 4, 6, 8, 9, 10 feet)	Exclusion of mobility disability (inability to ambulate with or without assistive devices)	Clinical diagnosis/DSM-IV and III R

Verghese et al. (84)	MC	3,128	≥60 years	Self-report	Instrumented walkway (GAITRite)/timed walk	Exclusion of mobility disability (inability to ambulate with or without assistive devices)	BIMC, MMSE

Allali et al. (85)	CS	314	≥65 years	CDR/GDS*/AD8	Instrumented walkway (GAITRite)	Preserved ADL (82)	Clinical and neuropsychological information

Doi et al. (86)	CS	9,683	≥65 years	GDS*	Timed walk (over 6.4 m)	Independent in basic ADL	MMSE

*Slow gait = one SD or more below age- and sex-appropriate mean values established in the same cohort*.

CS, community setting; MC, multiple cohorts; *N*, number of subjects; CERAD, Consortium to Establish a Registry for Alzheimer’s Disease questionnaire; GDS, Geriatric Depression Scale; GDS*, “*Do you feel you have more problems with memory than most?” A positive response (“yes”) on the question was used to define subjective memory complaints*; DSM-(III/IV R), Diagnostic and Statistical Manual of Mental Disorders (revised third or fourth edition criteria); AD8, cognitive screening instrument; CDR, clinical dementia rating; ADL, activities of daily living; IADL, instrumental activities of daily living; BIMC, blessed information-memory-concentration; MMSE, Mini-mental State Examination.

Motoric cognitive risk was found to have a prevalence ranging from 2 to 18% (83, 84, 86, 87). A multi-country (17 countries worldwide) study showed an overall pooled prevalence of almost 10% (83). These studies have shown MCR to have a higher prevalence and incidence in older age irrespective of gender. Based on the current availability of data, cross country comparison lowest prevalence of 2% was found in the United Kingdom and Australian cohort, and higher prevalence of 15% in Indian cohort and the highest prevalence of 16–18% was found in French population (83, 84, 86, 87).

Factors such as stroke, diabetes, obesity, depression, and sedentariness have been found to be associated with high risk of MCR in older adults (85, 86). Additional studies in much diverse sociodemographic settings are required for confirming the global burden of the condition and accordingly identify the associated risk factors.

### MCR Syndrome As a Predictor Of

#### Dementia

Relevant links between cognition and gait have been established earlier. Older adults with cognitive impairment are known to have slower pace (26). Verghese and colleagues in the early 2000 implicated that presence of neurological gait in older adults could predict the risk of dementia (18). However, predictive capacity of MCR with regards to subtypes of dementia was found to be different according to study cohort. In the Einstein Aging Study, MCR was found to be highly prevalent with age and was a strong predictor of vascular dementia (VaD) (19). Older subjects with MCR were found to be at more than 3-folds risk [hazard ratio (HR) = 3.27] of future dementia (except AD) and particularly over 12-folds risk (HR = 12.81) of VaD. However, slow gait was the only gait parameter used which might have decreased the predictive validity of MCR.

In another multi-country study, MCR predicted dementia in multiple cohorts as well as pooled sample, with risks ranging from 1.79- to 2.10-folds (83). Interestingly, MCR was found to be associated with increased risk of AD in two cohorts of the study with 2.21- and 1.97-folds risk, while very few cases of VaD dementia were present in the cohort. However, the cohorts were limited to only 17 countries; therefore, the predictive strength cannot represent for all at-risk subjects [as the primary criteria of MCR cognitive complaint and slow gait can vary demographically (88)], and not to forget the major risk factors that have varied demographic distribution. Additionally, information on APOE-4 genotype [that is known to impact progression of dementia (55)] was not included on this multi-country study, which could have further strengthened the validity of MCR dementia predictive capacity.

Interestingly, a retrospective study in Japanese older population has further elaborated the relation between MCR and dementia (89). The authors have reported the rate of conversion to dementia was 119.8/1,000 persons per year in MCR population, while the non-MCR group was 102.5/1,000 persons per year (OR = 1.38). Slow gait and low scores in executive function tests were found to be predictive of higher rate of conversion to dementia.

#### Falls

A very high frequency of falls (32–42% per year) in older people over 70 years has been estimated (90), which could result in many detrimental effects including disability or death (91, 92). As discussed earlier, maintaining a normal well-balanced gait requires an efficient integration of motoric, cognitive, and psychological function (3, 5, 16, 17) and the inability to maintain a normal gait could result in falls. Impairment in cognitive domains such as executive function, attention, processing speed, and memory is known to increase the risks of falls. However, age-related loss in white matter integrity is thought to be one of the key mechanisms affecting the cognitive domain responsible (57, 93). Thus, from our explanations earlier, we could expect MCR to be a sensitive predictor of falls in older adults.

A study by Callisaya and colleagues with a combined five large cohorts across three countries found subjects with MCR to be at 44% at high risk of falls in pooled analysis (21). The study showed that slow gait [risk ratio (RR) = 1.30] and memory complaints (RR = 1.25) were also individually associated with increased risk of falls. Whereas, exclusion of MCR case in the study showed a slight decreased association of slow gait (RR = 1.25) and memory complaints (RR = 1.17) with falls. Even after adjustments for previous falls, MCR was significantly associated with falls (RR = 1.29) and multiple falls (RR = 1.37) in pooled analysis. No doubt, the results from the study show MCR to be an effective risk screening tool for falls, as the associations observed were relatively stronger. However, due to different criteria/procedures used for diagnosis of MCR and falls, heterogeneity was present in the pooled analysis.

#### Disability

Very few studies discussing the associations of MCR and disability are available. However, it can be expected that older individuals with coexisting memory decline and physical limitation are likely to be disabled (or lose independency) if not provided with proper medical attention at an early phase. A very recent study involving 4,235 Japanese older adults (mean age of 72 years) has suggested MCR to be able to predict risk for disability (HR = 1.69) (22). The diagnostic criterion for disability was here regarded as certification by long-term care insurance. Nevertheless, the study has provided some perspectives for future studies, which could implement a more clinical diagnostic method for disability. The findings from this study have verified that individuals with slow gait and cognitive impairment are at high risk of disability, and more studies are demanded to confirm the findings.

#### Death

As discussed earlier, maintaining intact gait is a complex process requiring multisystem/multifunction coordination, therefore could represent a person’s holistic level of healthiness. Studies have shown gait speed to be a very strong predictor of survival (8) and cognitive impairment is also known to predict mortality (94). MCR involves both cognition and gait, has a high prevalence (83, 84, 86, 87), is known to predict falls (21), dementia (18, 19, 22), and therefore could be a more sensitive predictor of death.

A study by Ayers and Verghese (1) including 11,867 participants from three different cohorts found MCR at baseline was associated with increased overall mortality (HR = 1.69) and increased risk of death even after adjustments for gait and memory test scores (HR = 1.19). The results from the study showed MCR to be a predictor of 2 years mortality, but MCR death predictive capacity in dementia subjects was found to be insignificant. However, it should be noted that the included cohorts of the study were from Europe and United states; therefore, results from the study cannot be interpreted as the global mortality predictive capacity of MCR. Moreover, the study population were community dwelling older adults, who tend to be in better shape compared to the institutionalized. As the study was a population-based study cases of diagnosed dementia could have been underreported, which could have underestimated the prediction of mortality in diagnosed dementia subjects.

## The Paradox of MCR Syndrome

An existing paradox of the MCR is whether to consider it as a condition to treat or a mere matter for research purposes? As discussed above, MCR was found to predict wide spectrum of abnormalities in older adults (1, 19, 21, 22) and has a high prevalence ranging up to 18% (83, 84, 86, 87). For these reasons, attention on further approach of this novel syndrome is necessary. Although past studies have stressed on the versatility of diagnosing MCR (20), the clinical approach of the syndrome is vague. In the clinical scenario, we could only attempt to investigate the underlying pathologies of the condition and treat them in traditional manner (that requires various medical tests, despite of considering MCR easy to diagnose), which might require more resources and perhaps even create additional confusion in clinicians. On the other hand, if we consider it as screening tool to identify the at-risk population for the abnormalities it can predict, various effective tools for the purpose already exist (with prior extensive studies involving different sociodemographic population settings). In this context, MCR might be more suitable as a research entity to exclusively investigate the interactions between the physical and cognitive domains (which is not well understood yet) in older population with high risk of conditions that can be predicted by it. Furthermore, these understandings could enable us to design preventive strategies to ameliorate many geriatric conditions including dementia.

## Conclusion

In summary, with population aging, the burden of cognitive disorders such as dementia is still escalating. Frequent coexistence of physical limitation and cognitive decline occur in aging individuals, leading to many detrimental effects. MCR includes the evaluation of potential precursors of physical limitation (i.e., gait speed) and cognitive decline (i.e., subjective memory complaints) in order to predict multiple age-related conditions including dementia. Future perspective of MCR might be as a research tool to particularly investigate the relationship between physical and cognitive domain in older adults, further elucidate our understanding of the interaction between these two domains. Results from such studies could facilitate in designing more effective preventive intervention strategies against dementia and other age-related negative outcomes.

## Author Contributions

JC: manuscript preparation and drafting; all authors: conception and design, critical revision, and approvement for final version.

## Conflict of Interest Statement

The authors declare that the research was conducted in the absence of any commercial or financial relationships that could be construed as a potential conflict of interest. The reviewer AC and handling editor declared their shared affiliation.
